# Assessment of interrelationships between cognitive performance, symptomatic manifestation and social functioning in the acute and clinical stability phase of schizophrenia: insights from a network analysis

**DOI:** 10.1186/s12888-023-05289-4

**Published:** 2023-10-24

**Authors:** Błażej Misiak, Patryk Piotrowski, Jerzy Samochowiec

**Affiliations:** 1 Department of Psychiatry, Wroclaw Medical University, Pasteura 10 Street, Wroclaw, 50-367 Poland; 2 Department of Psychiatry, Pomeranian Medical University, Szczecin, Poland

**Keywords:** Psychosis, Cognition, Social functioning, Network analysis, Psychopathology

## Abstract

**Background:**

It has been shown that various aspects of clinical manifestation of schizophrenia are strongly related to social functioning. However, it remains unknown as to whether similar factors predict social functioning at various stages of psychosis. Therefore, the present study aimed to compare the effects of interconnections between various domains of psychopathology and neurocognition on social functioning in people during acute phase of psychosis and those during remission of positive and disorganization symptoms using a network analysis.

**Methods:**

Two independent samples of individuals with schizophrenia spectrum disorders were enrolled (89 inpatients during acute phase and 90 outpatients during remission of positive and disorganization symptoms). Clinical assessment covered the levels of functioning, positive, negative and depressive symptoms. Cognition was recorded using the Repeatable Battery for the Assessment of Neuropsychological Status (RBANS). Data were analyzed by means of the network analysis. Two separate networks of clinical symptoms, social functioning, and cognition (i.e., in patients during acute phase of psychosis and remitted outpatients with schizophrenia) were analyzed and compared with respect to the measures of centrality (betweenness, closeness, strength, and expected influence) and edge weights.

**Results:**

In both networks, the majority of centrality metrics (expected influence, strength, and closeness) had the highest values for the RBANS scores of attention (the sum of scores from two tasks, i.e., digit span and coding) and immediate memory. In both networks, social functioning was directly connected to positive, negative and depressive symptoms as well as the RBANS scores of attention and language. Additionally, in remitted patients, social functioning was directly connected to the RBANS score of immediate memory.

**Conclusions:**

Findings from the present study indicate the central role of cognitive deficits, especially those related to attention, processing speed, working and immediate memory in shaping functional impairments regardless of schizophrenia phase. Therapeutic interventions that aim to improve functional capacity need to target these domains of neurocognitive performance.

**Supplementary Information:**

The online version contains supplementary material available at 10.1186/s12888-023-05289-4.

## Introduction

Schizophrenia is a neurodevelopmental disorder that often leads to lasting impairments of social functioning [1]. It is ranked among the most important causes of disability worldwide [2]. Nevertheless, there is evidence that social functioning of people with psychotic disorders tends to improve over time, especially in those with lower baseline severity of negative symptoms and greater baseline quality of life [3]. This effect is more pronounced in people with shorter illness duration. At this point, it is important to note that recovery in schizophrenia should not only include remission of clinical symptoms but also improvement of social functioning that manifests in sustained employment, independent living, overall involvement in the society, and social interactions [4]. However, functional recovery rates are still low and reach about 58% in people with first-episode psychosis (FEP) [5] and about 38% in those with multiple-episode schizophrenia [6]. In this regard, the recognition of factors contributing to functional recovery holds important implications for clinical practice.

It is important to note that impairments of social functioning in schizophrenia are largely attributable to all aspects of psychopathological manifestation, including positive and negative symptoms, formal thought disorder as well as affective symptoms [7, 8]. Furthermore, it has been shown that impairments of neurocognition serve as an important factor contributing to decreased social functioning of people with schizophrenia [9]. Neurocognitive impairments observed in people with schizophrenia affect several domains, including speed of processing, attention/vigilance, working memory, verbal learning, visual learning as well as reasoning and problem solving [10–12]. Moreover, there is evidence that individuals with schizophrenia show deficits of social cognition defined by a range of processes that enable to interpret social contexts and respond appropriately [13]. A recent meta-analysis of studies performed in people with early psychosis demonstrated that impairments across all domains of neurocognitive performance and social cognition are associated with lower psychosocial functioning both cross-sectionally and longitudinally [14]. However, there is evidence that neurocognitive deficits might affect social functioning through the effects on social cognition [15–17]. Other factors that have been associated with lower social functioning include earlier age of psychosis onset [18] and longer duration of untreated psychosis [19].

Neurocognitive deficits in people with schizophrenia are closely related to negative symptoms. The National Institute of Mental Health (NIMH) developed the Measurement and Treatment Research to Improve Cognition in Schizophrenia (MATRICS) initiative that aimed to develop the consensus about the definition and methods of measuring negative symptoms [20]. According to the NIMH-MATRICS consensus statement, negative symptoms include avolition, anhedonia, asociality, blunted affect, and alogia [20–22]. Negative symptoms are now perceived as the core component of schizophrenia that largely contributes to long-term disability and poor functional outcomes [23].

Our understanding of psychopathological manifestation of schizophrenia, its underlying mechanisms, and clinical relevance has largely improved with the development of network approaches to data analysis. A network analysis assumes that mental disorders represent systems of causally associated symptoms as opposed to the effects of a single latent factor [24]. This approach to analysis of psychopathology allows to address its complexity without indicating a specific model [25]. Findings are represented by networks, where specific variables (nodes) are connected with edges. Due to the possibility to indicate central nodes of psychopathology, i.e., those that have the highest number of strong connections with other nodes, a network analysis holds the usefulness to indicate the most important targets for therapeutic interventions.

To date, some studies have aimed to address the complexity of connections between various aspects of clinical manifestation of schizophrenia that are of importance for shaping social functioning. For instance, Galderisi et al. [26] found that improving functional capacity and daily life skills might be critical for planning any therapeutic interventions. Another study investigated differential effects of interrelationships between psychopathology, metacognition, and neurocognition on functional capacity in people with early- and late-stage of schizophrenia [27]. The authors found that the global network structure and strength do not differ significantly between both stages of schizophrenia. Moreover, this study demonstrated that visual learning and disorganization symptoms serve as the most central determinants of social functioning in schizophrenia. Also, disorganization and negative symptoms as well as metacognition appeared to be directly and strongly associated with real-life functioning. Investigating determinants of social functioning at various stages of schizophrenia might be of importance for potential personalization of therapeutic approaches. However; these aspects have not been thoroughly addressed by the prior studies. In other words, it remains unknown as to whether there are the same symptomatic and neurocognitive determinants of social functioning during the acute relapse and clinical stability phase of schizophrenia. To address this research gap, the present study aimed to perform and compare separate network analyses of psychopathology, neurocognition, and social functioning in individuals with acute relapse of schizophrenia spectrum disorders and those during clinical stability phase.

## Methods

### Participants

Two independent samples of individuals with schizophrenia spectrum disorders (total *n* = 208) were analyzed in the present study. This was a secondary analysis of samples enrolled in different projects. In both samples, the diagnosis was based on the DSM-IV criteria and confirmed using Operational Criteria for Psychotic Illness (OPCRIT) checklist [28].

The first sample included individuals during acute phase of psychosis (*n* = 89). All of them were enrolled at inpatient units. Among them, there were 42 individuals with FEP and 47 individuals during acute relapse of schizophrenia. The following diagnoses were established in subjects with FEP: schizophrenia (*n* = 16), schizoaffective disorder (*n* = 5), schizophreniform disorder (*n* = 7), brief psychotic disorder (*n* = 13), and delusional disorder (*n* = 1). The majority of individuals with FEP (*n* = 40, 95.2%) were medicated on the day of enrollment; however, the total treatment duration did not exceed 30 days.

The second sample included outpatients with schizophrenia during remission of positive and disorganization symptoms (*n* = 90) according to the criteria developed for the Positive and Negative Syndrome Scale (PANSS) [29–31]. In other words, all patients from this sample scored ≤ 3 on the PANSS items P1 (“delusions”), P2 (“conceptual disorganization”), P3 (“hallucinatory behavior”), N5 (“difficulty in abstract thinking”), G5 (“mannerism and posturing”), G9 (“unusual thought content”), and G11 (“poor attention”). Moreover, all of them received a stable regimen of antipsychotics over the period of at least 6 preceding months (no dosage adjustment > 25% occurred during this time period). The study was conducted in accordance with the Declaration of Helsinki. The protocol of this study was approved by the Bioethics Committees at Wroclaw Medical University (Wroclaw, Poland) and Pomeranian Medical University (Szczecin, Poland). All patients provided written informed consent for participation in the present study and for the use of their anonymized data for research purposes.

### Assessments

In both samples, symptomatic manifestation and social functioning were recorded using PANSS [29] and the Social and Occupational Functioning Assessment Scale (SOFAS) [32]. The PANSS score of negative symptoms was calculated as the sum of scores from the following items: N1 – blunted affect, N2 – emotional withdrawal, N3 – poor rapport, N4 – passive/apathetic social withdrawal, and N6—lack of spontaneity and flow of conversation [22]. Scores from the items N5 – difficulty in abstract thinking and N7 – stereotyped thinking were not included as they do not represent negative symptoms. Depressive symptoms were assessed using the Montgomery-Asberg Depression Rating Scale (MADRS) [33] in the sample of individuals during acute phase of psychosis and the Calgary Depression Scale for Schizophrenia (CDSS) [34] in the sample of individuals during clinical stability.

Cognitive performance was examined by means of the Repeatable Battery for the Assessment of Neuropsychological Status (RBANS) [35]. The RBANS is composed of 12 tasks that measure performance of immediate memory (list learning and story memory), visuospatial/constructional abilities (figure copy and line orientation), language (picture learning and semantic fluency), attention (digit span and coding), and delayed memory (list recall, list recognition, story recall, and figure recall). Higher RBANS scores refer to better cognitive performance.

### Data analysis

First, general characteristics of both samples were compared using the χ^2^ test and the Mann–Whitney U test or t-tests (depending on data distribution). Normality of data distribution was assessed using the Kolmogorov–Smirnov test. This part of the data analysis was performed in the SPSS software, version 25.0. The level of significance was set *p* < 0.05.

Next, we performed and plotted two separate network analyses, i.e., in participants during acute phase of psychosis and those during clinical stability. The following variables (represented by network nodes) were used: 1) the PANSS score of positive symptoms; 2) the PANSS score of negative symptoms; 3) the MADRS or CDSS score; 4) the SOFAS score, and 5) the RBANS scores. The *EBICglasso* was used to estimate the network. In order to reduce the number of false-positive of false positive edges, we used the least absolute shrinkage and selection operator (LASSO) [36]. This approach allows to shrink low-weight edges by assigning the zero value to them. The following measures of node centrality were further calculated and plotted: betweenness, closeness, strength, and expected influence. Betweenness can be defined as the number of times a node lies on the shortest pathway between any other two nodes. Closeness shows how close a node is located to all other nodes in the network. In turn, strength refers to the sum of edge weights connected to a specific node. Finally, expected influence is a relatively new measure that shows the strength of a node’s influence within the network accounting for the presence of negative edges [37].

The case-drop bootstrapping with 1,000 iterations was performed in order to assess stability of centrality measures and edge weights. To analyze network stability, we plotted average correlations of centrality metrics and edge weights with the percentage of cases retained after the case-drop procedure. These plots allow to indicate the correlation stability coefficient (CS-C). The CS-C refers to maximum percentage of cases that can be dropped from the data to retain, with 95% probability, a correlation of at least 0.7 between statistics from the original network and statistics computed with a lower number of cases. The acceptable CS-C value should be at least 0.25 [38]. The network accuracy was analyzed using non-parametric bootstrapping with 1,000 iterations. Closer means of bootstrapped edge weights to original edge weights together with lower 95%CI correspond with greater accuracy. The network estimation as well as the analysis of centrality metrics and the measures of robustness (stability and accuracy) were carried out in the JASP software, version 0.17.

The final part of data analysis was related to the comparison of networks in patients during acute phase of psychosis and those during clinical stability. We compared the following aspects of both networks: 1) edges; 2) global strength and 3) strength and expected influence of specific nodes. The Benjamini–Hochberg correction was applied to account for multiple testing. This analysis was based on the network comparison test R package [39]. This package includes a series of permutation tests developed for Gaussian and binary data using invariance measures.

## Results

### Descriptive characteristics of the samples

Both samples of patients did not differ significantly in terms of sex, the number of education years, employment status, cognitive performance, and the majority of metrics related to pharmacotherapy (Table 1). As expected, the levels of positive and negative symptoms as well as impairment of social functioning were significantly greater in patients during acute phase of psychosis. The dosage of antipsychotics, expressed as chlorpromazine equivalents, was significantly higher in the group of patients during clinical stability.

**Table 1:** Descriptive characteristics of the sample

	Acute phase, *n* = 89	Clinical stability, *n* = 90	p
Age, years	36.7 ± 13.5	43.5 ± 12.0	** < 0.001**
Sex, females	41 (46.1)	35 (38.9)	0.331
Education, years	13.1 ± 2.3	13.7 ± 3.5	0.108
Employed or student	35 (39.3)	27 (30.0)	0.190
PANSS, positive symptoms	14.0 ± 5.1	5.1 ± 1.9	** < 0.001**
PANSS, negative symptoms	15.9 ± 9.0	10.2 ± 5.0	** < 0.001**
CDSS	–	1.8 ± 3.0	–
MADRS	8.0 ± 8.1	–	–
SOFAS	45.0 ± 16.5	64.3 ± 19.0	** < 0.001**
RBANS, immediate memory	38.0 ± 11.0	38.0 ± 10.1	0.841
RBANS, visuospatial/constructional	32.4 ± 7.2	34.2 ± 5.3	0.169
RBANS, language	26.5 ± 6.5	25.6 ± 6.3	0.191
RBANS, attention	45.0 ± 15.4	44.8 ± 14.9	0.878
RBANS, delayed memory	42.8 ± 10.4	42.3 ± 9.6	0.751
Illness duration, years	7.5 ± 12.2	20.1 ± 13.1	** < 0.001**
FGAs	27 (30.3)	22 (24.4)	0.658
SGAs	69 (77.5)	74 (82.2)	0.433
Clozapine	15 (16.9)	20 (22.2)	0.365
CPZeq, mg/day	385.6 ± 212.9	593.3 ± 366.5	** < 0.001**
Antidepressants	7 (7.9)	12 (13.3)	0.235
Mood stabilizers	15 (16.9)	9 (10.0)	0.178

Significant differences (*p* < 0.05) are marked in bold

Data expressed as mean ± SD or n (%)

*Abbreviations*: *CDSS* the Calgary Depression Scale for Schizophrenia, *CPZeq* Chlorpromazine equivalents, *FGAs* First-generation antipsychotics, *MADRS* the Montgomery-Asberg Depression Rating Scale, RBANS the Repeatable Battery for the Assessment of Neuropsychological Status, *SGAs* Second-generation antipsychotics

### Network analysis

**Fig. 1:**
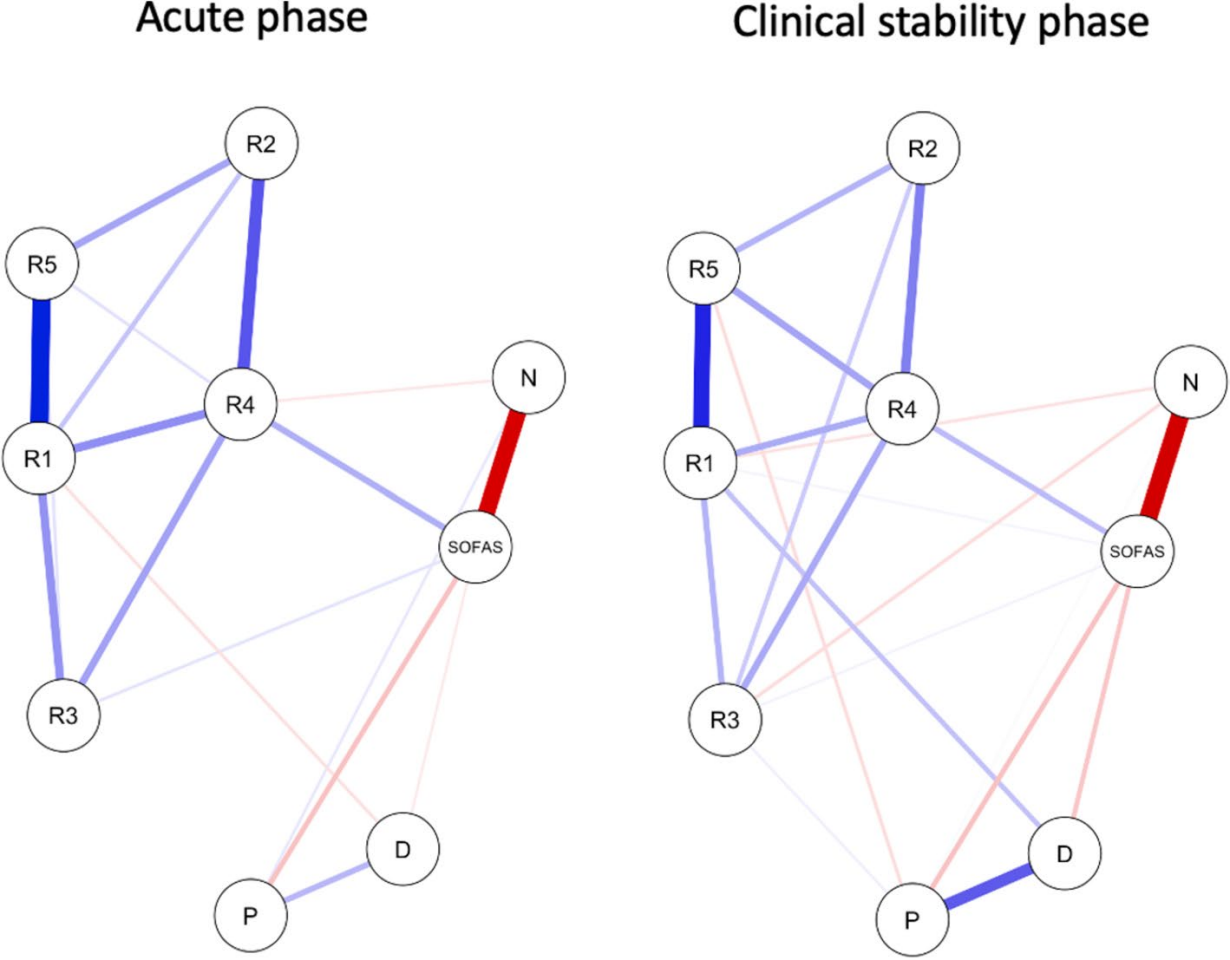
The networks analyzed in the present study. Specific variables are presented as nodes that are connected with edges. Thicker edges correspond with greater edge weights. Blue edges show positive associations, while red edges depict negative associations. Abbreviations: D, depressive symptoms; N, negative symptoms; P, positive symptoms; R1, immediate memory; R2, visuospatial/constructional abilities; R3, language; R4, attention; R5, delayed memory; SOFAS, the Social and Occupational Functioning Assessment Scale

**Fig. 2:**
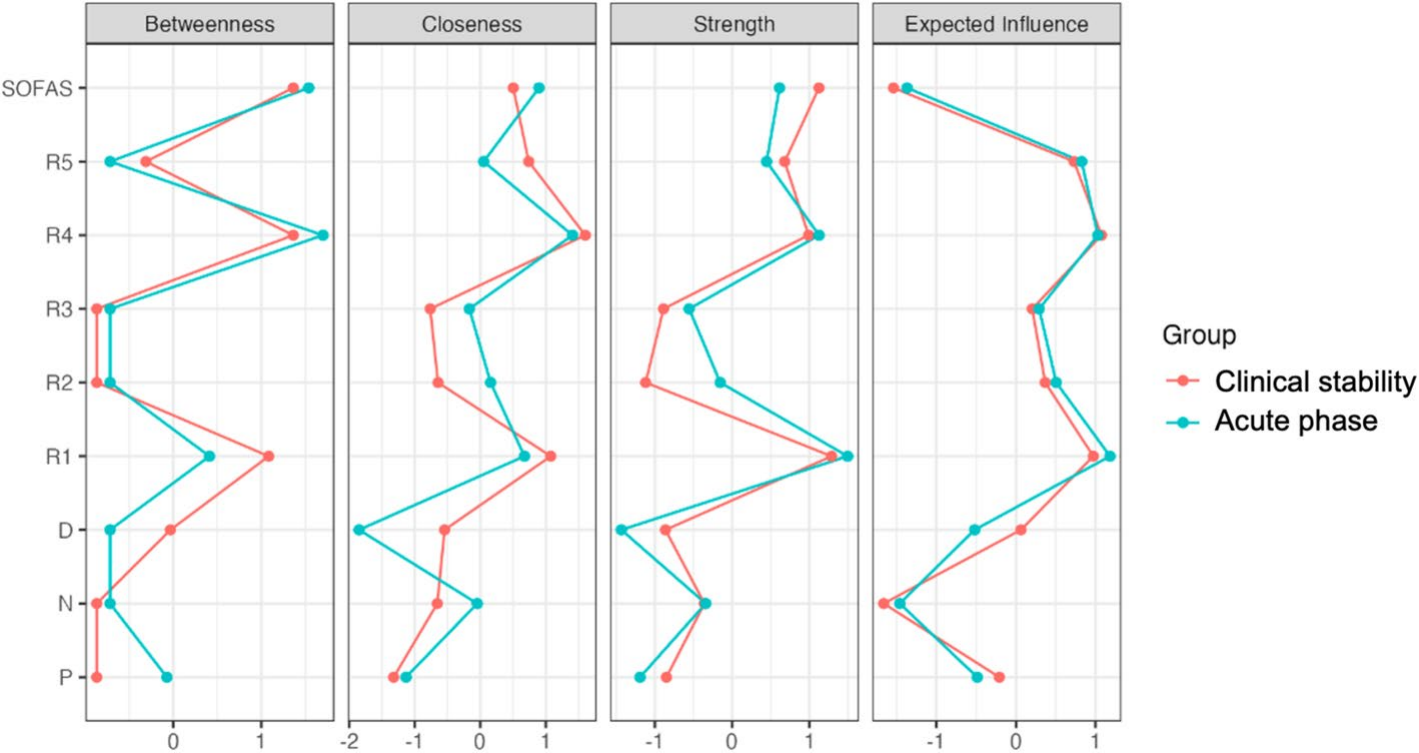
The node centrality measures. Separate plots illustrate various measures of node centrality including betweenness, closeness, strength, and expected influence. Abbreviations: D, depressive symptoms; N, negative symptoms; P, positive symptoms; R1, immediate memory; R2, visuospatial/constructional abilities; R3, language; R4, attention; R5, delayed memory; SOFAS, the Social and Occupational Functioning Assessment Scale

All nodes appeared to be well connected (Fig. 1). Out of 36 potential edges, there were 21 non-zero edges in remitted patients (58.3%) and 18 non-zero edges in patients during the acute phase of psychosis (50.0%) (see Table S1 for edge weights). In patients during clinical stability, the node representing the SOFAS score was directly connected to nodes of positive symptoms (edge weight = –0.142), negative symptoms (edge weight = –0.612), depressive symptoms (edge weight = –0.130), immediate memory (edge weight = 0.027), language (edge weight = 0.030), and attention (edge weight = 0.160). In turn, in patients during acute phase of psychosis, the following nodes were directly connected to the SOFAS score: positive symptoms (edge weight = –0.108), negative symptoms (edge weight = –0.421), depressive symptoms (edge weight = –0.034), language (edge weight = 0.044), and attention (edge weight = 0.144). These observations indicate that the largest edge weight for direct connections with the SOFAS score was obtained for the attention score in both networks. The node centrality measures were plotted in Fig. 2 (see also Table S2). The majority of them indicated the highest centrality values for the RBANS scores of attention and immediate memory in both networks.

The CS-C values for edges and centrality metrics were greater than 0.25 indicating sufficient stability of the network (Figs. S1 and S2). The bootstrapped means of edge weights and the original edge weights were almost overlapping and the 95%CI values of edge weights were relatively narrow. These observations indicate sufficient accuracy of the network analyses (Fig. S3).

Both networks did not differ significantly in the network invariance test (test statistic M = 0.264, *p* = 0.220) and the centrality invariance test (test statistic C = 0.390, *p* = 0.266). However, global strength was significantly higher in the sample of remitted patients compared to the sample of acutely relapsed patients (3.828 vs. 2.651, test statistic S = 1.176, *p* = 0.006). However, there were no significant differences with respect to specific node centrality metrics between both samples (Table S3). Edge weights did not differ significantly, except for the edge weight for the connection between depressive symptoms and immediate memory that appeared to positive in remitted patients and negative in acutely relapsed patients (0.139 vs. –0.050, *p* < 0.001, Table S4).

## Discussion

Findings from the present study indicate that associations of clinical manifestation with functional capacity are similar at various stages of schizophrenia with important roles of positive, negative and depressive symptoms as well as neurocognitive impairments. This observation is in agreement with results of the prior study showing no significant differences in the network of factors related to social functioning between early- and late-stage schizophrenia [27]. Also, the network comparison of remitted and non-remitted individuals with schizophrenia has demonstrated that depressive, positive, and negative symptoms are linked in a stable way [40]. In our study, impairments of neurocognition appeared to be the most central predictor of social functioning. This observation was demonstrated for the RBANS score of immediate memory (only in remitted patients with schizophrenia) and attention (in both samples) that is composed of scores from two tasks, i.e., digit span and symbol coding. Apart from attention, these tasks measure other aspects of cognitive performance including processing speed and working memory, respectively. Finally, our study confirmed that all domains of psychopathology are associated with social functioning in people with schizophrenia regardless of illness stage [7]. Although in our study both samples were similar in terms of sociodemographic characteristics (except for age) and the level of cognitive performance, some differences need to be pointed out. Indeed, acutely relapsed patients had higher levels of positive and negative symptoms as well as lower levels of social functioning. Moreover, the dosage of antipsychotics was significantly higher in remitted patients with schizophrenia.

Our observations about the central role of neurocognition in shaping functional capacity are in agreement with the prior studies. For instance, a prospective study of individuals with FEP, based on a network analysis, revealed that working memory appears to have the highest centrality in predicting social functioning [41]. Another cross-sectional study of stable outpatients with schizophrenia demonstrated that working memory deficits measured using the letter number sequencing task were among the most central nodes predicting social functioning [42]. Also, the study based on 81 individuals with schizophrenia spectrum disorders in a non-acute phase revealed the greatest centrality of the PANSS cognitive factor score and working memory. However, the authors did not assess social functioning [43]. In turn, the study by Karyakina and Shmukler [44] compared the networks of neurocognitive performance in people with schizophrenia spectrum disorders and healthy controls. The authors found that processing speed impairments, measured using the symbol coding task, were among the most central neurocognitive impairments in people with schizophrenia spectrum disorders. Among healthy controls, working memory appeared to have the greatest centrality among all neurocognitive domains. Processing speed and working memory impairments represent the most important aspects of cognitive deficits observed in psychotic disorders that can already be detected in people at clinical high risk of psychosis (especially those who transit to overt psychosis over time) [45], first-degree relatives of people with schizophrenia [46], and individuals with FEP [47].

The central role of attention, processing speed, immediate and working memory indicates that these domains of neurocognition should be the target for interventions that also aim to improve social functioning in schizophrenia. Among these interventions, several studies have focused on cognitive remediation. The most recent meta-analyses revealed that cognitive remediation strategies are effective in improving cognitive performance with low-to-moderate effect size estimates [48, 49]. Interestingly, one of these meta-analyses, that synthesized evidence from studies performed among inpatients with schizophrenia, suggested efficacy in improving processing speed, memory, and working memory [48]. However, evidence with respect to improvement of social and global functioning was less reliable. At this point, it is important to note that cognitive remediation strategies use various protocols and target various domains of neurocognitive functioning. In this regard, our findings might provide additional support for selecting targets of cognitive remediation.

There are some limitations of our study. First, our sample size was not large. However, a network analysis achieved sufficient stability and accuracy, likely due to relatively low number of nodes included in the network. Second, insights into social functioning might be limited due to the use of SOFAS that does not provide insights into various aspects of social functioning. Also, the PANSS does not provide comprehensive insights into the current conceptualization of negative symptoms and fails to fails to assess subject’s internal experience. Third, a network analysis did not analyze other important predictors of social functioning, e.g., those related to social cognition and metacognition. Nevertheless, it is important to note that impairments of social cognition and metacognition might be the consequence of neurocognitive deficits [15–17]. Therefore, it is likely that inclusion of these variables would not change considerably the centrality measures. Fourth, our sample of individuals during acute phase of psychosis was not homogenous in terms of specific diagnostic categories. Finally, although we used a network analysis approach, a lack of prospective design does not allow to provide solid insights into causality.

In sum, findings from the present study indicate that the effects of clinical manifestation on social functioning in schizophrenia spectrum disorders do not differ considerably between acute and clinical stability phases. Importantly, our observations indicate that impairments of attention, processing speed as well as working and immediate memory might be the most central predictors of social functioning in schizophrenia spectrum disorders. This gives a translational perspective indicating that interventions aimed at improving functional capacity of individuals with schizophrenia should focus on these domains of neurocognitive performance.

### Supplementary Information

**Additional file 1:** **Table S1.** Edge weights (1 – stable outpatients; 2 – patients during the acute phase of psychosis). **Table S2.** Strength centrality measures (1 – stable outpatients; 2 – patients during the acute phase of psychosis). **Table S3.** The comparison of strength and expected influence between both networks (p-values after the Benjamini-Hochberg correction are shown). **Table S4. **The comparison of edge weights between both samples. **Figure S1. **Stability of centrality metrics. Average correlations with original sample is ploted against the percentage of sampled cases. The maximum percentage of cases that can be dropped from the data to retain, with 95% probability, a correlation of at least 0.7 between statistics from the original network and statistics computed with a lower number of cases should be at least 0.25 (the CS-C value). **Figure S2.** Stability of edge weights. The maximum percentage of cases that can be dropped from the data to retain, with 95% probability, a correlation of at least 0.7 between statistics from the original network and statistics computed with a lower number of cases should be at least 0.25 (the CS-C value). **Figure S3.** Bootstrapped 95% confidence intervals of estimated edge weights. The grey area shows bootstrapped 95% confidence intervals. Red points refer to edge weights in the sample, whole black points depict bootstrapped edge weights.

## Data Availability

The datasets used and analysed during the current study are available from the corresponding author on reasonable request.
