# Supplementary material for: Assessment of interrelationships between cognitive performance, symptomatic manifestation and social functioning in the acute and clinical stability phase of schizophrenia: insights from a network analysis
Source: BMC Psychiatry. 2023 Oct 24;23:774. doi: 10.1186/s12888-023-05289-4 (PMC10594890; doi:10.1186/s12888-023-05289-4)
Supplement: Supplementary file 1 — Additional file 1: Table S1. Edge weights (1 – stable outpatients; 2 – patients during the acute phase of psychosis). Table S2. Strength centrality measures (1 – stable outpatients; 2 – patients during the acute phase of psychosis). Table S3. The comparison of strength and expected influence between both networks (p-values after the Benjamini-Hochberg correction are shown). Table S4. The comparison of edge weights between both samples. Figure S1. Stability of centrality metrics. Average correlations with original sample is ploted against the percentage of sampled cases. The maximum percentage of cases that can be dropped from the data to retain, with 95% probability, a correlation of at least 0.7 between statistics from the original network and statistics computed with a lower number of cases should be at least 0.25 (the CS-C value). Figure S2. Stability of edge weights. The maximum percentage of cases that can be dropped from the data to retain, with 95% probability, a correlation of at least 0.7 between statistics from the original network and statistics computed with a lower number of cases should be at least 0.25 (the CS-C value). Figure S3. Bootstrapped 95% confidence intervals of estimated edge weights. The grey area shows bootstrapped 95% confidence intervals. Red points refer to edge weights in the sample, whole black points depict bootstrapped edge weights. [file 12888_2023_5289_MOESM1_ESM.docx]

**Supplementary Appendix**

**Table S1.** Edge weights (1 – stable outpatients; 2 – patients during the acute phase of psychosis).


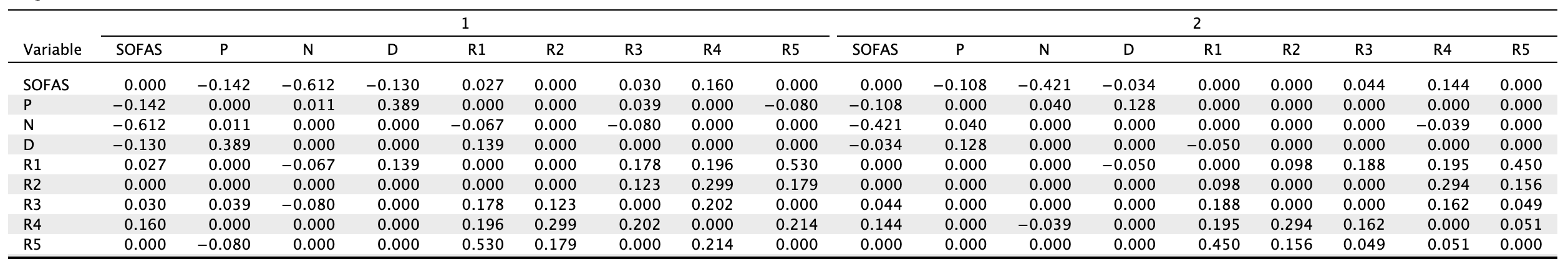


Abbreviations: D, depressive symptoms; N, negative symptoms; P, positive symptoms; R1, immediate memory; R2, visuospatial/constructional abilities; R3, language; R4, attention; R5, delayed memory; SOFAS, the Social and Occupational Functioning Assessment Scale.

**Table S2.** Strength centrality measures (1 – stable outpatients; 2 – patients during the acute phase of psychosis).


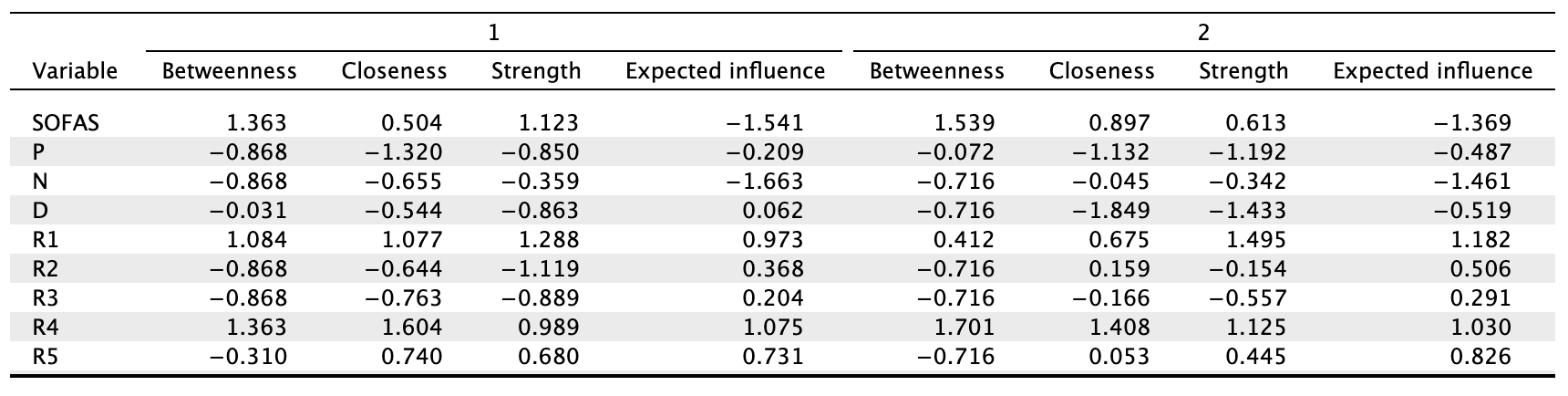


Abbreviations: D, depressive symptoms; N, negative symptoms; P, positive symptoms; R1, immediate memory; R2, visuospatial/constructional abilities; R3, language; R4, attention; R5, delayed memory; SOFAS, the Social and Occupational Functioning Assessment Scale.

**Table S3.** The comparison of strength and expected influence between both networks (p-values after the Benjamini-Hochberg correction are shown).

| Node | Strength | Expected influence |
| --- | --- | --- |
| P | 0.276 | 0.653 |
| N | 0.354 | 0.342 |
| D | 0.270 | 0.342 |
| R1 | 0.435 | 0.508 |
| R2 | 0.813 | 0.813 |
| R3 | 0.355 | 0.813 |
| R4 | 0.454 | 0.342 |
| R5 | 0.342 | 0.610 |
| SOFAS | 0.276 | 0.342 |

Abbreviations: D, depressive symptoms; N, negative symptoms; P, positive symptoms; R1, immediate memory; R2, visuospatial/constructional abilities; R3, language; R4, attention; R5, delayed memory; SOFAS, the Social and Occupational Functioning Assessment Scale.

**Table S4.** The comparison of edge weights between both samples.

| Edges | p-value^*^ |
| --- | --- |
| P – N | 1.000 |
| P – D | 0.221 |
| N – D | 1.000 |
| P – R1 | 1.000 |
| N – R1 | 0.221 |
| D – R1 | < 0.001 |
| P – R2 | 1.000 |
| N – R2 | 1.000 |
| D – R2 | 1.000 |
| R1 – R2 | 0.724 |
| P – R3 | 0.881 |
| N – R3 | 0.060 |
| D – R3 | 1.000 |
| R1 – R3 | 1.000 |
| R2 – R3 | 0.432 |
| P – R4 | 1.000 |
| N – R4 | 0.221 |
| D – R4 | 1.000 |
| R1 – R4 | 1.000 |
| R2 – R4 | 1.000 |
| R3 – R4 | 1.000 |
| P – R5 | 0.054 |
| N – R5 | 1.000 |
| D – R5 | 1.000 |
| R1 – R5 | 1.000 |
| R2 – R5 | 1.000 |
| R3 – R5 | 0.789 |
| R4 – R5 | 0.785 |
| P – SOFAS | 1.000 |
| N – SOFAS | 0.221 |
| D – SOFAS | 1.000 |
| R1 – SOFAS | 0.616 |
| R2 – SOFAS | 1.000 |
| R3 – SOFAS | 1.000 |
| R4 – SOFAS | 1.000 |
| R5 – SOFAS | 1.000 |

^*^p-values after the Benjamini-Hochberg correction

Abbreviations: D, depressive symptoms; N, negative symptoms; P, positive symptoms; R1, immediate memory; R2, visuospatial/constructional abilities; R3, language; R4, attention; R5, delayed memory; SOFAS, the Social and Occupational Functioning Assessment Scale.

**Figure S1.** Stability of centrality metrics. Average correlations with original sample is ploted against the percentage of sampled cases. The maximum percentage of cases that can be dropped from the data to retain, with 95% probability, a correlation of at least 0.7 between statistics from the original network and statistics computed with a lower number of cases should be at least 0.25 (the CS-C value).

**
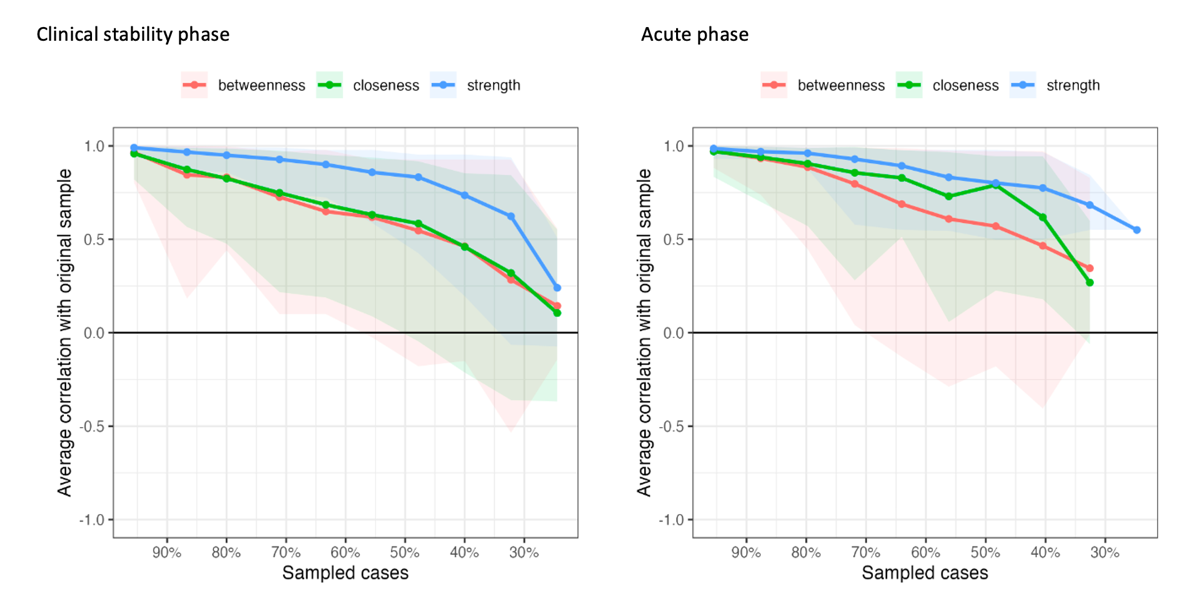
**

**Figure S2.** Stability of edge weights. The maximum percentage of cases that can be dropped from the data to retain, with 95% probability, a correlation of at least 0.7 between statistics from the original network and statistics computed with a lower number of cases should be at least 0.25 (the CS-C value).

**
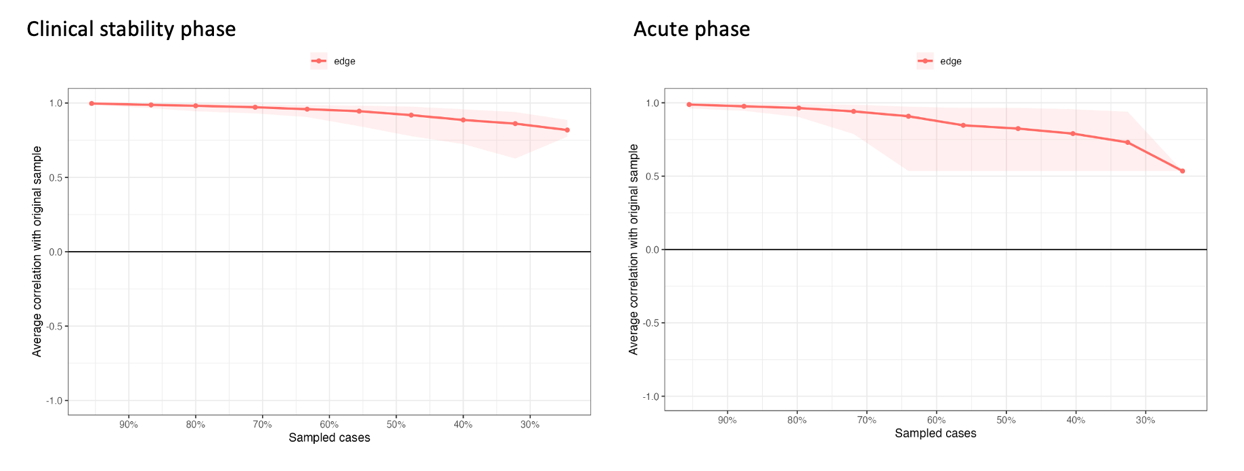
**

**Figure S3.** Bootstrapped 95% confidence intervals of estimated edge weights. The grey area shows bootstrapped 95% confidence intervals. Red points refer to edge weights in the sample, whole black points depict bootstrapped edge weights.


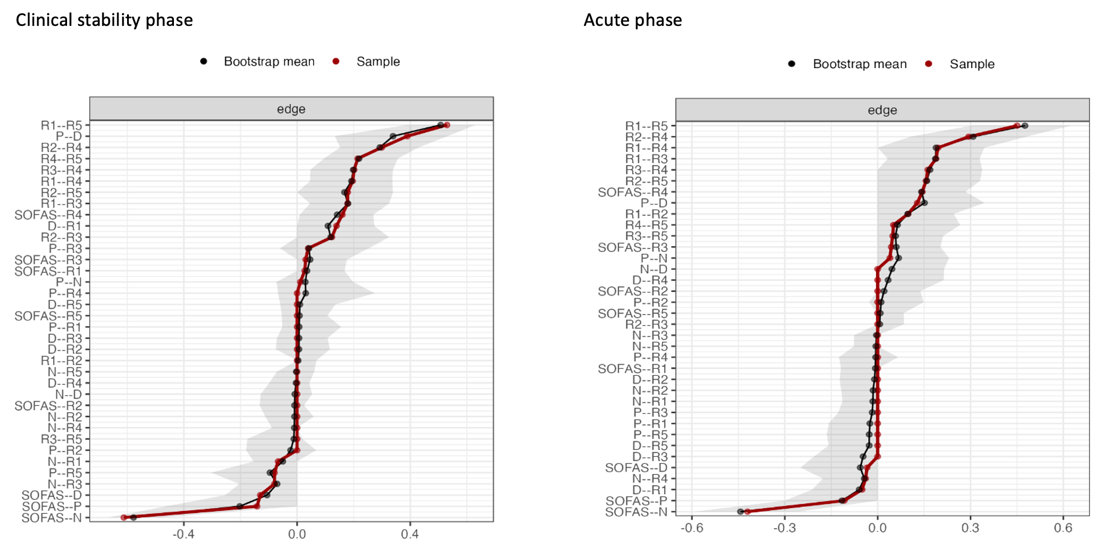


Abbreviations: D, depressive symptoms; N, negative symptoms; P, positive symptoms; R1, immediate memory; R2, visuospatial/constructional abilities; R3, language; R4, attention; R5, delayed memory; SOFAS, the Social and Occupational Functioning Assessment Scale.
